# Transcriptome and metabolite profiling reveals the effects of *Funneliformis mosseae* on the roots of continuously cropped soybeans

**DOI:** 10.1186/s12870-020-02647-2

**Published:** 2020-10-21

**Authors:** Cheng-Cheng Lu, Na Guo, Chao Yang, Hai-Bing Sun, Bai-Yan Cai

**Affiliations:** 1 Heilongjiang Provincial Key Laboratory of Ecological Restoration and Resource Utilization for Cold Region, College of Life Sciences, Heilongjiang University, Harbin, 150080 People’s Republic of China; 2 Department of Food and Environment Engineering, Heilongjiang East University, Harbin, 150086 People’s Republic of China

**Keywords:** Soybean root rot, *Funneliformis mosseae*, *Fusarium oxysporum*, Transcriptome, Metabolite profiling

## Abstract

**Background:**

Arbuscular mycorrhizal fungi are the most widely distributed mycorrhizal fungi, which can form mycorrhizal symbionts with plant roots and enhance plant stress resistance by regulating host metabolic activities. In this paper, the RNA sequencing and ultra-performance liquid chromatography (UPLC) coupled with tandem mass spectrometry (MS/MS) technologies were used to study the transcriptome and metabolite profiles of the roots of continuously cropped soybeans that were infected with *F. mosseae* and *F. oxysporum*. The objective was to explore the effects of *F. mosseae* treatment on soybean root rot infected with *F. oxysporum*.

**Results:**

According to the transcriptome profiles, 24,285 differentially expressed genes (DEGs) were identified, and the expression of genes encoding phenylalanine ammonia lyase (*PAL*), trans-cinnamate monooxygenase (*CYP73A*), cinnamyl-CoA reductase (*CCR*), chalcone isomerase (*CHI*) and coffee-coenzyme o-methyltransferase were upregulated after being infected with *F. oxysporum*; these changes were key to the induction of the soybean’s defence response. The metabolite results showed that daidzein and 7,4-dihydroxy, 6-methoxy isoflavone (glycine), which are involved in the isoflavone metabolic pathway, were upregulated after the roots were inoculated with *F. mosseae.* In addition, a substantial alteration in the abundance of amino acids, phenolic and terpene metabolites all led to the synthesis of defence compounds. An integrated analysis of the metabolic and transcriptomic data revealed that substantial alterations in the abundance of most of the intermediate metabolites and enzymes changed substantially under pathogen infection. These changes included the isoflavonoid biosynthesis pathway, which suggests that isoflavonoid biosynthesis plays an important role in the soybean root response.

**Conclusion:**

The results showed that *F. mosseae* could alleviate the root rot caused by continuous cropping. The increased activity of some disease-resistant genes and disease-resistant metabolites may partly account for the ability of the plants to resist diseases. This study provides new insights into the molecular mechanism by which AMF alleviates soybean root rot, which is important in agriculture.

## Background

Soybean (*Glycine max* L*.*) root rot is a kind of crop disease that is widely distributed, causes serious damage and is difficult to control. Its incidence can reach approximately 75% ~ 90%, which leads to declining soybean yields and quality [39]. The pathogenic fungi that cause soybean root rot include *Fusarium oxysporum*, *Fusarium avenaceum, Fusarium solanacearum*, *Fusarium merismoides*, *Phytophthora sojae* and *Pythium ultimum* [16]. *F. oxysporum* is the dominant fungus of soybean root rot, and it can reduce the number of soybean pods and the yields between 25% ~ 75% [57]. Studies have shown that the fungicides could control the disease incidence in a greenhouse experiment, but they had no effect on increasing production in the field [14].

Microorganisms have biocontrol potential against plant diseases (Babu et al., [2]). Arbuscular mycorrhizal fungi (AMF) are obligate mutualistic fungi, which can form mycorrhizal symbionts with the roots of over 80% of terrestrial plants. AMF can not only enhance plants’ absorption of nutrients and minerals, but also improve the ability of plants to resist soil-borne disease, and plays an important role in plant evolution and nutrition [7, 19, 36]. *Funneliformis mosseae* as one dominant AMF, has a positive effect on plant tolerance to root pathogens. In 1968, Safir first discovered that *F. mosseae* could reduce the incidence rate of onion (*Allium cepa* L.) root rot caused by *Pyrenochaeta terrestris* [42], and later investigators have successfully applied it to citrus (*Citrus reticulata Blanco*), peaches (*Amygdalus persica* L.), strawberries (*Fragaria ananassa* Duch*.*), soybeans and other crops. For example, the root rot caused by *F. oxysporum* in cucumber (*Cucumis sativa* L.) seedlings [47] and the root rot caused by *Meloidogyne incognita* and *Macrophomina phaseolina* in chickpeas [44] as well as aboveground plant diseases such as powdery mildew (*Erysiphe pisi*) in *Elymus sibircus* [10] and *Fusarium* wilt in cucumbers have been studied in this context [11].

The transcriptome can be used to reveal the mechanism of metabolic regulation at the molecular level, and it has become an indispensable method for studying gene expression, RNA biogenesis and metabolism [49, 50]. Metabolomics is an emerging omics technology, it is applied to identify and quantify all the metabolites in an organism or in cells, and is a component of systems biology [24]. This tool is a bridge to link genes, proteins, and phenotypes. Thus far, metabolomics has been gradually applied in various fields of agriculture and has been demonstrated to be a powerful tool to determine the metabolic profile of biological samples through targeted or untargeted analyses [22]. Agudeloromero combined transcriptome and metabolite analysed the response of grapes (*Vitis vinifera* L.) against the pathogen, which bring novel insights into the responses of fruits during a pathogen–host interaction [1]. By integrating proteome and metabolite profiling with cell wall properties, Floerl S et al. found that *Verticillium longisporum* might enhance its own pathogenicity by negatively regulating and delaying the induction and expression of plant defence genes [8].

Owing to the increased demand for soybeans and the enhanced risk of crop losses caused by *F. oxysporum*, it is necessary to find disease control ways that could be used in soybean production systems as soon as possible. The purpose of this study is to combine transcriptome and metabolite profiling to explore the effect of *F. mosseae* on soybean root rot through pot experiments and to determine whether AMF could alleviate the damage from *F. oxysporum-*derived soybean root rot. In addition, other purposes are to reduce the incidence of soybean root rot, alleviate the obstacles to continuous cropping, provide a test basis for elucidating the pathogenesis of soybean root rot, and provide a theoretical basis for the development and application of biological agents.

## Results

### Impact of *F. mosseae* treatments on the root rot grade and colonization rate of soybean roots

**Fig. 1:**
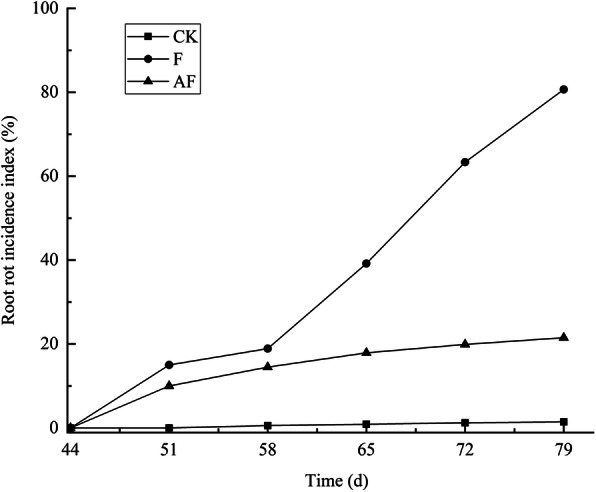
Root rot incidence index in potted-experiments of continuously cropped soybeans. Squares, circles, and triangles represent samples from the CK, F, and AF groups, respectively. CK means soybean were planted the normal sterilized soil; F means soybean seeds were inoculated with *F. oxysporum* spore suspension; AF means soybean seeds were inoculated with *F. mosseae* + *F. oxysporum*. Note: All experiments were conducted in potted-experiments. X-axis represents which day detected the root rot incidence index of soybean. Y-axis represents the root rot incidence index

Forty-four days after sowing, the roots of soybeans in each treatment group were randomly sampled to detect the incidence of root rot. Figure 1 shows that the disease index increased in F group, with the growth and development of the soybeans. After *F. mosseae* inoculation, the disease index of AF group was lower than that of F group, which indicated that *F. mosseae* could alleviate the symptoms of soybean root rot.

**Fig. 2:**
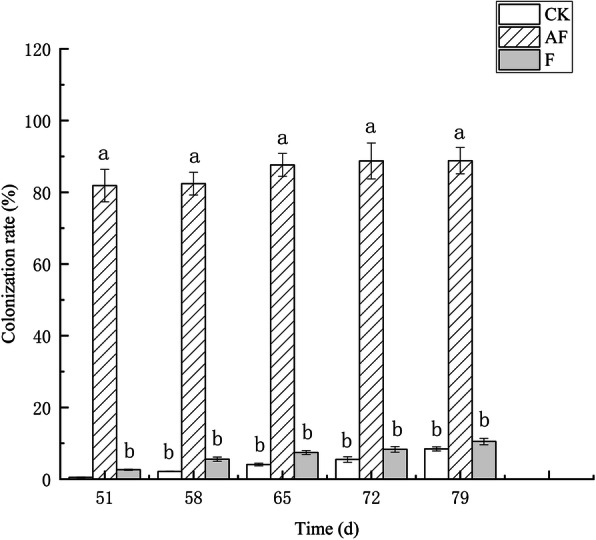
AMF colonization rate of continuously cropped soybeans in potted-experiments. White, gray and stripes represent samples from the CK, F, and AF groups, respectively. CK means soybean were planted the normal sterilized soil; F means soybean seeds were inoculated with *F. oxysporum* spore suspension; AF means soybean seeds were inoculated with *F. mosseae* + *F. oxysporum*. Note: All experiments were conducted in potted-experiments. X-axis represents which day detected the AMF colonization rate of continuously cropped soybean roots. Y-axis represents the AMF colonization rate. The data shown here are the averages for three plants under each condition and the error bars represent standard deviations. Bars subtended by the same lowercase letter do not differ significantly at *p* < 0.05 according to Tukey’s test

As shown in Fig. 2, the colonization rate of AMF was not detected until the soybeans had grown for 40 d, and the colonization rate increased in all the groups. The colonization rate in AF was significantly higher than it was in the CK and F groups (*N* = 2, *P* = 0.044). After 58 d, the mycorrhizal structure that formed in the AF group gradually increased, and a large number of hyphae and vesicles appeared. Clearly, the colonization rate of F and CK was significantly lower than that of AF. We speculate that the roots infected in these two groups were by the spore transmission of fungi spores in the air.

### Impact of *F. mosseae* treatments on the soybean root transcriptome

Nine root samples were transcriptome-sequenced during the high-incidence period of root rot. The number of high-quality clean reads accounted for more than 98% of the samples in each group (Supplementary Table S1). The high-quality clean reads obtained here were compared with known genomes. Last, 38,961, 38,832 and 38,688 genes were detected in the AF, CK and F treatments, and the number of genes detected in each group is shown in Table 1.

**Table 1:** Number of genes detected in each treatment

Group name	Number of known genes	Number of new genes	Total number of genes
AF	38,961 (82.91%)	2313	41,274
CK	38,832 (82.63%)	2298	41,130
F	38,688 (82.33%)	2284	40,792

**Fig. 3:**
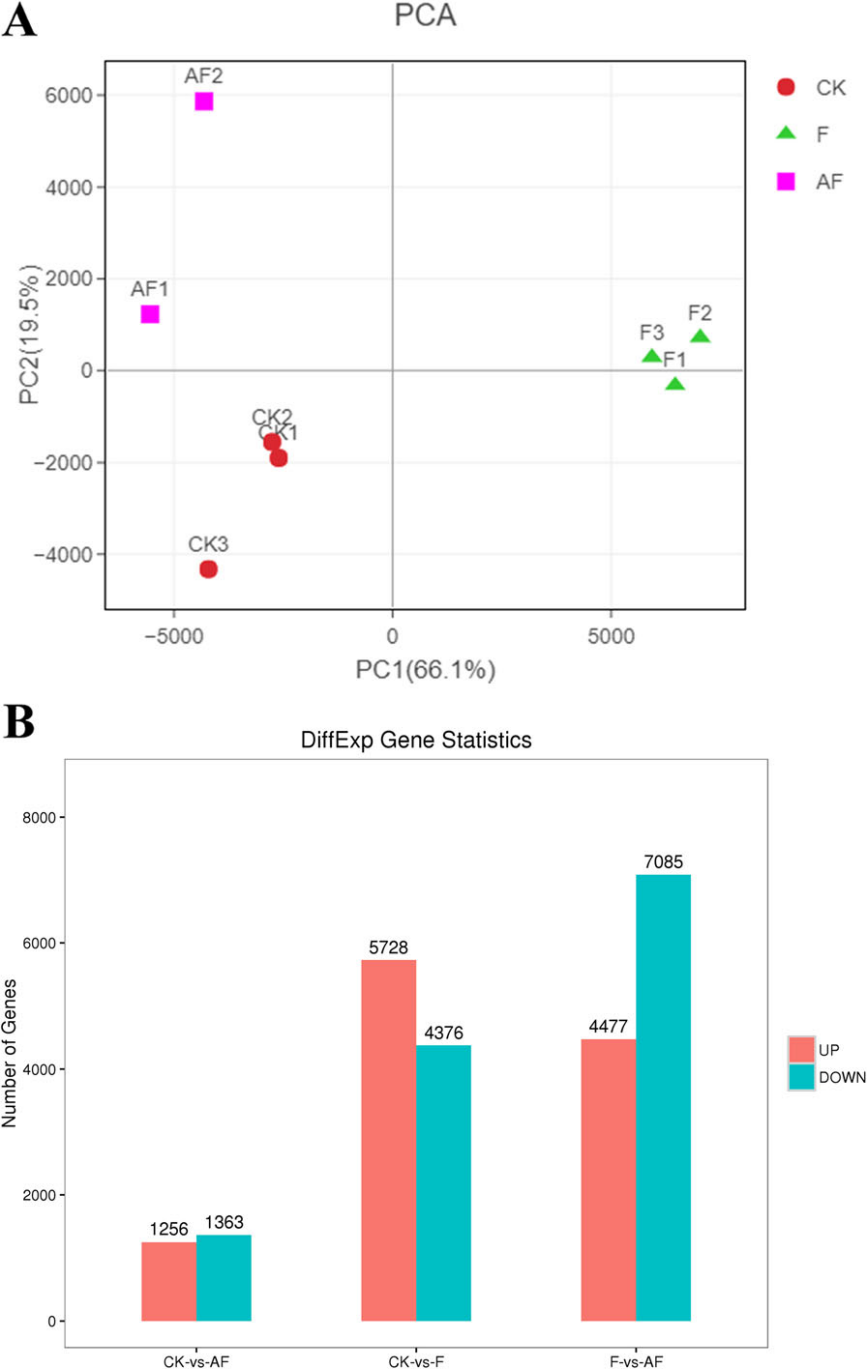
Transcriptome analysis of continuously cropped soybean roots during the high incidence of soybean root rot. **a**. Principal component analysis (PCA) on the soybean transcriptome of 9 independent samples collected from soybean roots. Circles, triangles and squares represent samples from the CK, F, and AF groups, respectively. **b**. Principal component analysis (PCA) between samples after removing the discrete sample AF3. **c**. Statistical histogram of differentially expressed genes in continuously cropped soybean roots between different groups. FDR < 0.05 and |log2FC| > 1 were used to screen differentially expressed genes. The red bar represents the percentage of upregulated genes, and the green bar represents the percentage of downregulated genes

To assess the reproducibility of soybean DEGs library, a principal component analysis was performed on the transcriptome profiles of the 9 analysed samples. The dispersion degree of F group was the best in PC1 and PC2, followed by CK group. Although the dispersion degree of CK3 was relatively large, its dispersion was within a reasonable range on PC1. In the AF group, AF3 become an outlier. To improve the repeatability among the three samples, the PCA analysis of each component was performed again after AF3 was removed (Fig. 3a). It can be seen that the dispersion degree of each component on PC1 (66.1%) is almost consistent, and the samples show good repeatability. After RNA-seq sequencing, to express CK, F and AF quantitatively, edge R software was used to analyse the DEGs between CK, F and AF groups (Fig. 3b). The different expression patterns among the three groups revealed that the difference between the F and AF groups was the largest (4477 downregulated transcripts and 7085 upregulated transcripts). In addition, when comparing CK and F, 5728 transcripts were upregulated and 4376 were downregulated.

**Fig. 4:**
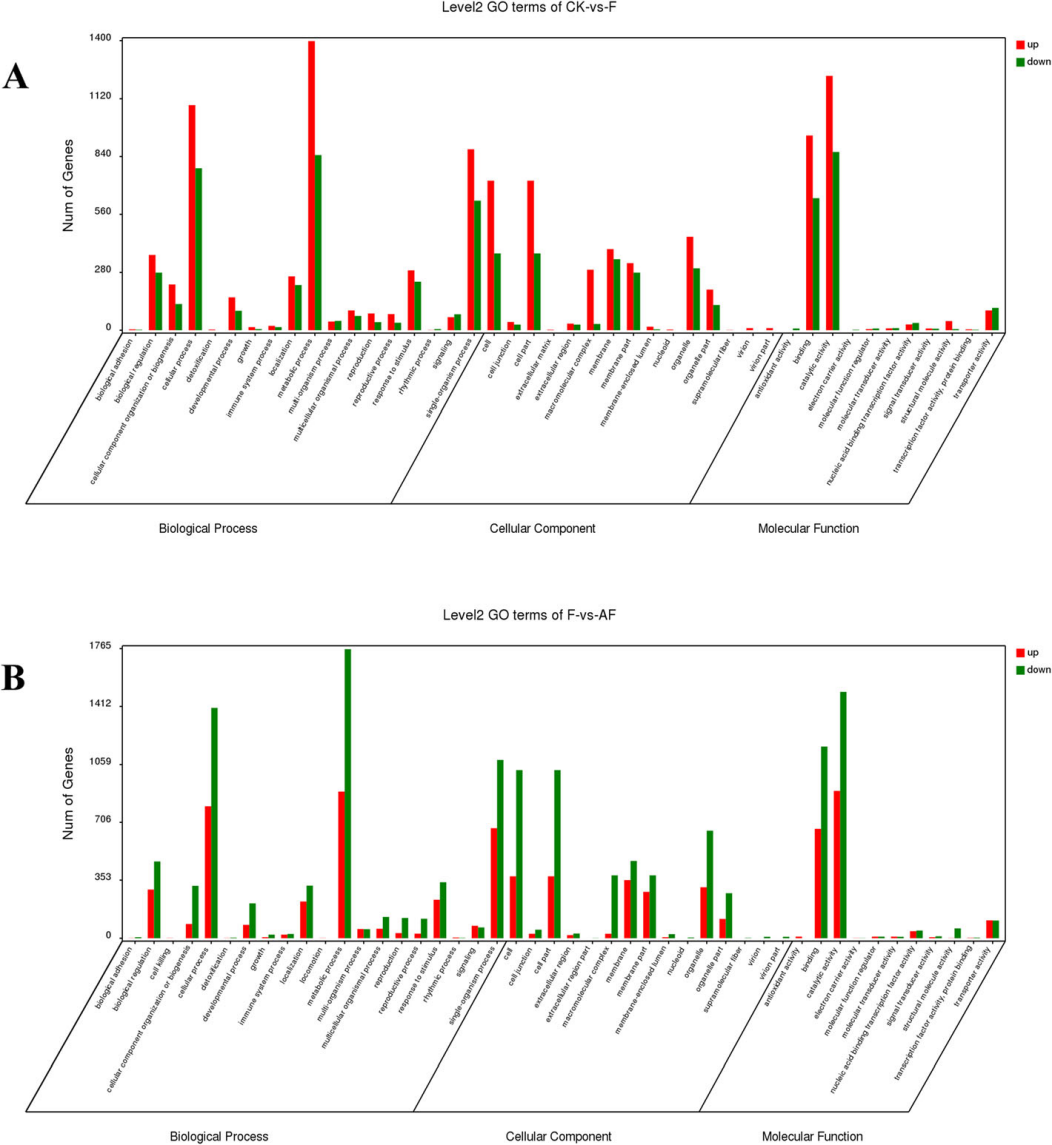
GO distribution of transcripts in continuously cropped soybean roots with different treatments during the high incidence of root rot. GO categories that were significantly enriched were analysed for their level of significance in a pair-wise comparison (A: CK vs. F and B: F vs. AF). The transcripts were annotated into three primary categories, namely, cellular component, biological process and molecular function. The x-axis indicates different GO terms. The y-axis represents the number of genes in the indicated categories. The red bar represents the percentage of upregulated genes, and the green bar represents the percentage of downregulated genes

We annotated and classified DEGs from three aspects: biological process (BP), molecular function (MF) and cellular component (CC) according to Gene Ontology. The GO terms of the DEGs in the CK vs. F groups were categorized into 44 primary functional groups (Fig. 4a). In the BP category, most DEGs were primarily concentrated on metabolic processes (12.67%) and cellular processes (10.57%). In the MF category, DEGs were primarily involved in coding catalytic activity (11.80%), followed by binding activity (8.92%). In the CC category, the upregulated DEGs were largely related to cell part (12.36%) and organelle (4.24%), while a few DEGs are involved in extracellular matrix (0.01%) and supramolecular fibers (0.005%). These results indicate that pathogens invade the cells of the soybean roots by destroying the membrane system, and then they disrupt the metabolic process of the soybeans and a series of physiological and biochemical reactions.

The GO terms of the DEGs were categorized into 46 primary functional groups in the F vs. AF groups (Fig. 4b). In the BP category, most DEGs were also primarily concentrated in the response process to stimulus (2.74%) and single-organism processes (8.39%), in additional to metabolic processes (12.68%) and cellular processes (10.54%). In the MF category, DEGs were primarily involved in coding catalytic activity (11.45%), followed by binding activity (8.76%). In the CC category, most of the DEGs were involved in coding organelle part (6.47%), cell components (9.94%) and cell membrane composition (7.04%), while few DEGs were associated with the extracellular region (0.23%) and supramolecular fibres (0.01%). After the *F. mosseae* inoculation, the DEGs were not only concentrated in GO terms related to the membrane system but also in GO terms related to the growth and development of soybeans, detoxification, antioxidants, etc., from the GO classification level, revealing the growth-promoting effect of *F. mosseae* on the soybeans.

**Fig. 5:**
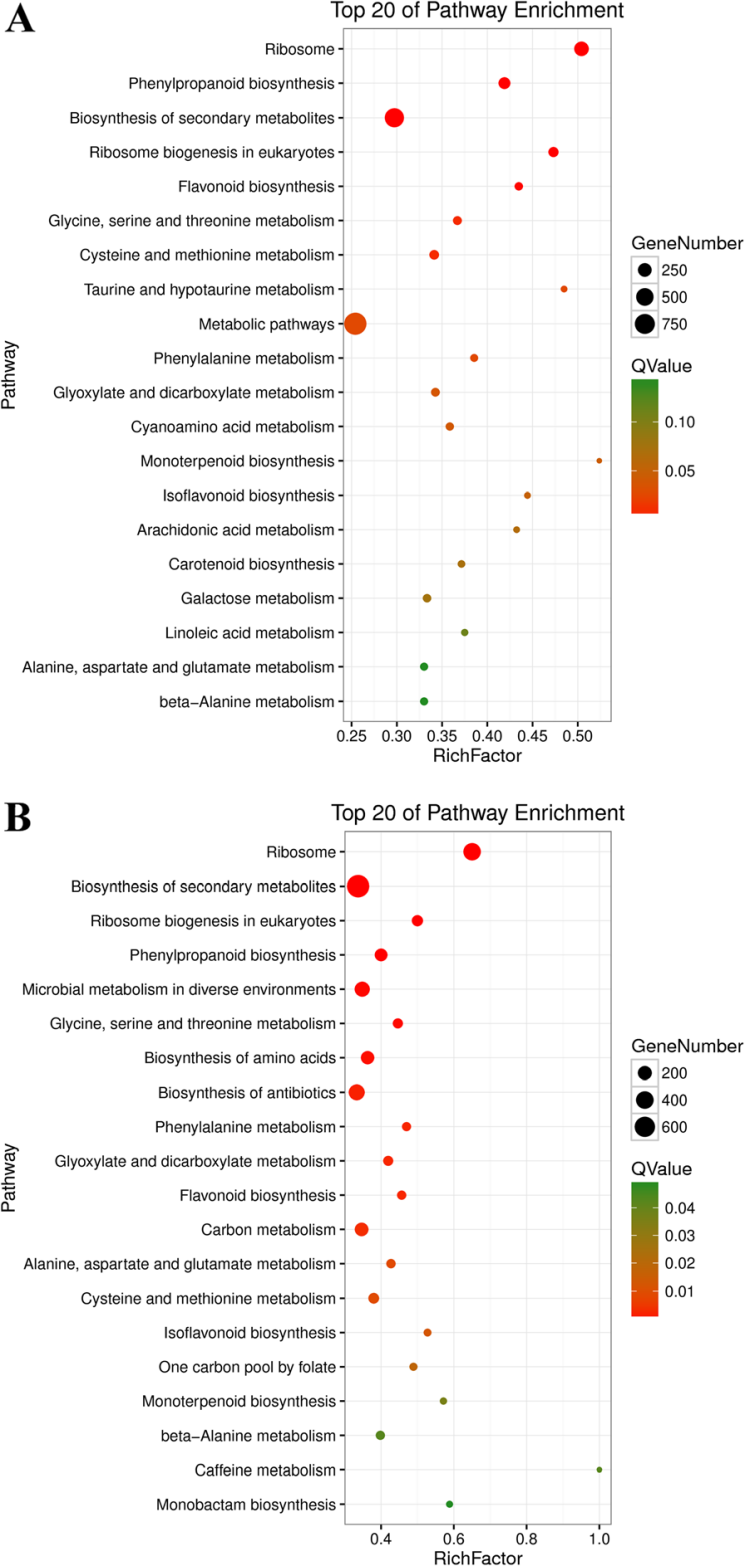
Statistical scatter diagram of KEGG pathway enrichment in CK vs. F A and F vs. AF B. The size of the points represents the number of differentially expressed genes, and different colours represent different Q-values. The larger the rich factor is, the higher the degree of enrichment. The Q-value is the *P*-value after multiple hypothesis testing and correction. The value range is 0 to 1. The closer to zero the number is, the more significant the enrichment

Instead of performing their functions independently, genes always coordinate with each other and perform a series of regulatory functions. Using the identified soybean root genes as the background, a KEGG enrichment analysis of significantly different genes can further clarify the functions of genes in metabolic pathways. The DEGs in the CK vs. F and F vs. AF groups were enriched in 131 and 132 KEGG metabolic pathways, respectively. With a *p*-value < 0.05 and an FDR < 0.05, metabolic and signal transduction pathways with significant changes were identified, and the top 20 metabolic pathways of the CK vs. F and F vs. AF groups are visually displayed by scatter diagram (Fig. 5).

Among the 131 pathways shown for CK vs. F (Supplementary Table S2), the three containing the highest numbers of DEGs were “metabolic pathways” (990 DEGs, 42.45%), “biosynthesis of secondary metabolites” (669 DEGs, 28.69%) and “ribosome” (304 DEGs, 13.04%). Other GO terms associated with high numbers of DEGs were “phenylpropanoid biosynthesis” (155 DEGs, 6.65%) and “plant-pathogen interaction” (104 DEGs, 4.46%). We found that genes involved in plant pathogen interactions, such as pathogenesis-related protein 1, mitogen-activated protein kinase kinase 1, and heat shock protein 90 kDa beta, were upregulated. In addition, genes encoding trans-cinnamate monooxygenase (CYP73A), cinnamyl-CoA reductase (CCR), and phenylalanine ammonia lyase (PAL) were also upregulated in the phenylpropanoid biosynthesis pathway, which indicated that the *F. oxysporum* infection induced the defence response in the soybeans.

Among the 132 pathways shown for F vs. AF (Supplementary Table S3), those containing the highest numbers of DEGs were “metabolic pathways” (1133 DEGs, 41.49%), “biosynthesis of secondary metabolites” (758 DEGs, 27.76%) and “ribosome” (392 DEGs, 14.35%). In addition, other GO terms associated with high numbers of DEGs were “biosynthesis of antibiotics” (303 DEGs, 11.09%) and “carbon metabolism” (185 DEGs, 6.77%). It is worth noting that genes that control the synthesis of phenylpropane, such as *PAL*, *CYP73A*, *CCR*, and coffee-coenzyme o-methyltransferase, showed a downregulated trend, in contrast to the CK vs. F group. The same is true of genes that control chalcone isomerase (*CHI*) synthesis in the flavonoid biosynthesis pathway and genes involved in the plant-pathogen interaction pathway. Therefore, it can be speculated that after AMF infection, the expression of *F. mosseae*-induced resistant enzyme genes were upregulated, and the continuous cultivation disease was alleviated by the action of *F. mosseae*, so the originally upregulated gene displayed the opposite expression trend.

### Impact of *F. mosseae* treatments on metabolite profiling

The multi-response monitoring (MRM) mode showing the multi-peak metabolite detection diagram (Supplementary Fig. S1) displays the substances that can be detected in the sample. The OPLS-DA model was used to screen which metabolites had significant changes (Supplementary Fig. S2). CK vs. F group and F vs. AF group scored 41 and 46 respectively in PC1. The dispersion of components was good in each group and there was a strong correlation between samples. Owing to multivariate statistical analysis sometimes showed overfitting, OPLS-DA models were further verified by cross validation and permutation test. According to VIP ≥ 1.0 and |p (corr)| > 0.5 to the OPLS-DA model and *p* value < 0.05 to the t-test, a total of 622 different metabolites were detected, as shown in Table 2, with 29 classes, including organic acids (76), amino acid derivative (53), hydroxycinnamoyl derivatives (38), isoflavones (17), benzoic acid derivatives (14), terpenoids (4), catechin derivatives (4) and other disease-resistant metabolites, allelochemicals and signalling molecules.

**Table 2:** Metabolite identification results

Type	Number	Percentage (%)
Organic acids	76	12.219
Nucleotide and derivates	61	9.807
Amino acid derivatives	53	8.521
Flavone	40	6.431
Hydroxycinnamoyl derivatives	38	6.109
Lipids_Glycerophospholipids	34	5.466
Amino acids	30	4.823
Lipids_Fatty acids	23	3.698
Carbohydrates	21	3.376
Flavonol	21	3.376
Flavanone	19	3.055
Lipids_Glycerolipids	18	2.894
Isoflavone	17	2.733
Vitamins	17	2.733
Anthocyanins	14	2.251
Benzoic acid derivatives	14	2.251
Flavone C-glycosides	13	2.090
Coumarins	12	1.929
Phenolamides	12	1.929
Alcohols and polyols	9	1.447
Indole derivatives	9	1.447
Quinate and its derivatives	8	1.286
Nicotinic acid derivatives	5	0.804
Alkaloids	4	0.643
Catechin derivatives	4	0.643
Cholines	4	0.643
Terpenoids	4	0.643
Tryptamine derivatives	4	0.643
Pyridine derivatives	3	0.482
Flavonolignan	2	0.322
Others	33	5.305

We combined a multivariate statistical analysis of the VIP ≥ 1 from the OPLS-DA and a univariate statistical analysis of the t-test *p*-value < 0.05 to screen the significant differences in metabolites between different comparison groups [41]. The results for the metabolites with significant differences are shown in Table 3. When comparing group CK and group F, 11 metabolites were upregulated and 22 were downregulated. Compared with AF, the number of upregulated and downregulated metabolites in F was 56 and 35.

**Table 3:** Statistics on the differential metabolites

Group	Up	Down	All
CK-vs-F	11	22	33
F-vs-AF	19	20	39
CK-vs-AF	16	14	30

**Fig. 6:**
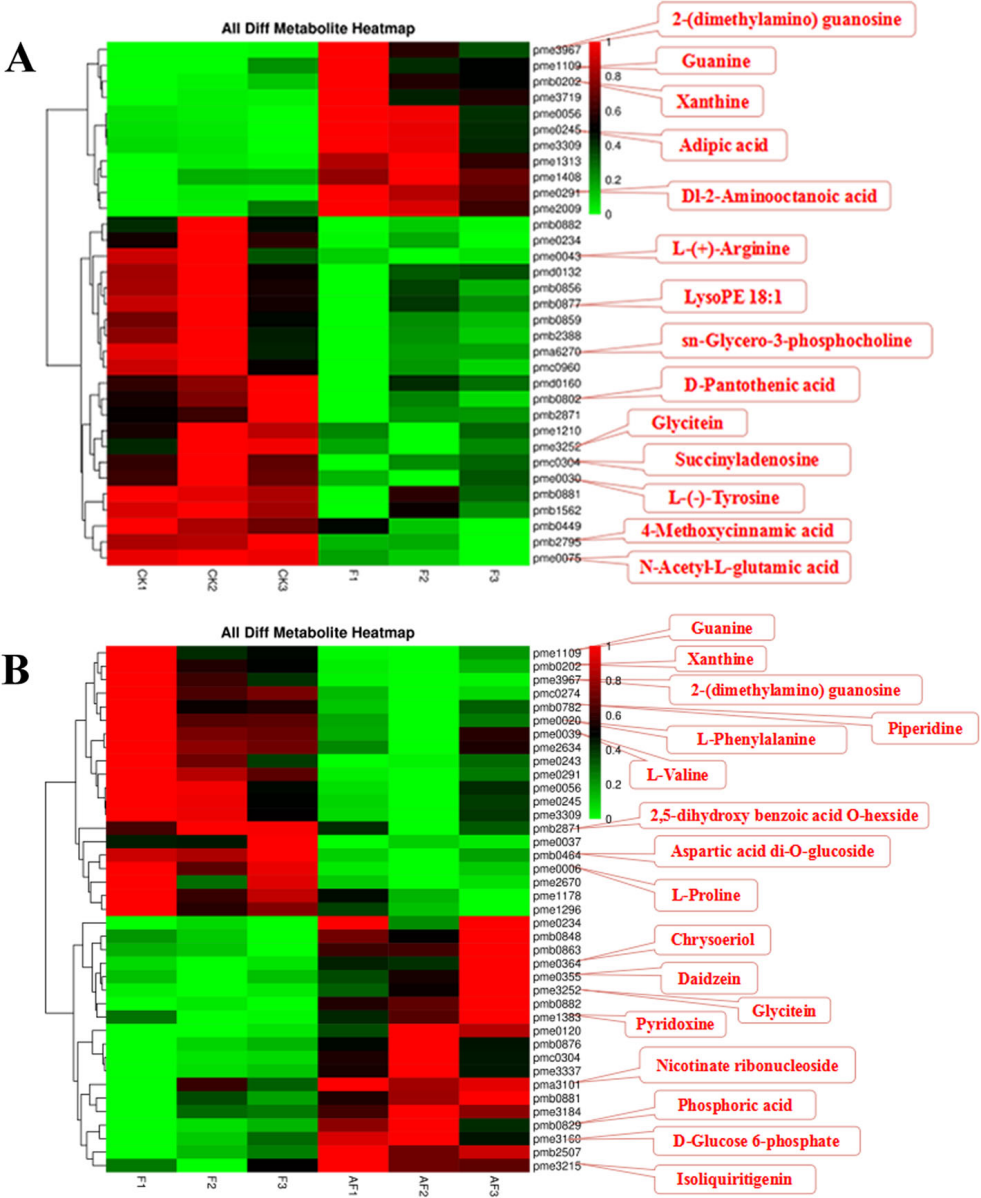
Differential metabolite cluster heat maps of continuously cropped soybean roots during the high incidence of root rot. (A): CK-vs-F; (B): F-vs-AF. Each row in the figure represents a different metabolite, and each vertical column represents a component, with colours representing the different intensity of the metabolites from red to green to indicate the intensity of the difference from large to small

A cluster analysis and heat map were created after the data normalization of the different metabolites between the comparison groups, which could visually show the accumulation difference in the differential metabolites between the groups (Fig. 6). There were 30 different metabolites, including nicotinic acid and its derivatives, flavonoids, and benzoic acid and its derivatives. Many of these metabolites have been shown to be effective in the plant defence response, including terpenoids and phenylpropanoids [13, 51].

All the differential metabolites were enriched in metabolic pathways to obtain differential metabolic pathways. CK vs. F showed 38 enriched metabolic pathways, and they were significantly enriched in amino acid biosynthesis (42.86%), metabolic pathways (78.57%), secondary metabolite biosynthesis (57.14%), monomer biosynthesis (14.29%), acetaldehyde and dicarboxylic acid metabolism (14.29%). During arginine and proline metabolism、tyrosine metabolism and arginine biosynthesis, the expression of arginine and tyrosine in Group F was significantly downregulated. In addition, the expression of pantothenic acid in Group F was also downregulated during the biosynthesis of CoA and pantothenic acid.

A total of 38 metabolic pathways were enriched in F vs. AF, with significant enrichment in purine metabolism (20.83%), secondary metabolite biosynthesis (37.5%), isoflavone biosynthesis (8.33%), glucoside biosynthesis (8.33%), lysine degradation (8.33%), fatty acid degradation (4.17%), pantothenic acid and CoA biosynthesis (4.17%), phenylalanine metabolism (4.17%) and other pathways. The expression of D-glucose 6-phosphate in AF group was upregulated during the metabolism of phosphoinositol. The expression of 4-hydroxy-2-quinolinic acid in AF group was upregulated in the tryptophan metabolism pathway. In addition, during isoflavone metabolic pathway, daidzein and 7,4-dihydroxy, 6-methoxy isoflavone (glycine) in AF group were also significantly upregulated.

From these findings, we can observe that the pathogen infection reduced the expression of organic acids and other metabolites and amino acid content in the soybean roots, resulting in serious plant disease and poor development. *F. mosseae* can induce and promote the expression of plant defence mechanisms and growth regulators, increase crop resistance, and be conducive to crop growth.

### Impact of *F. mosseae* treatments on integrated metabolites and transcript networks in soybean roots

**Fig. 7:**
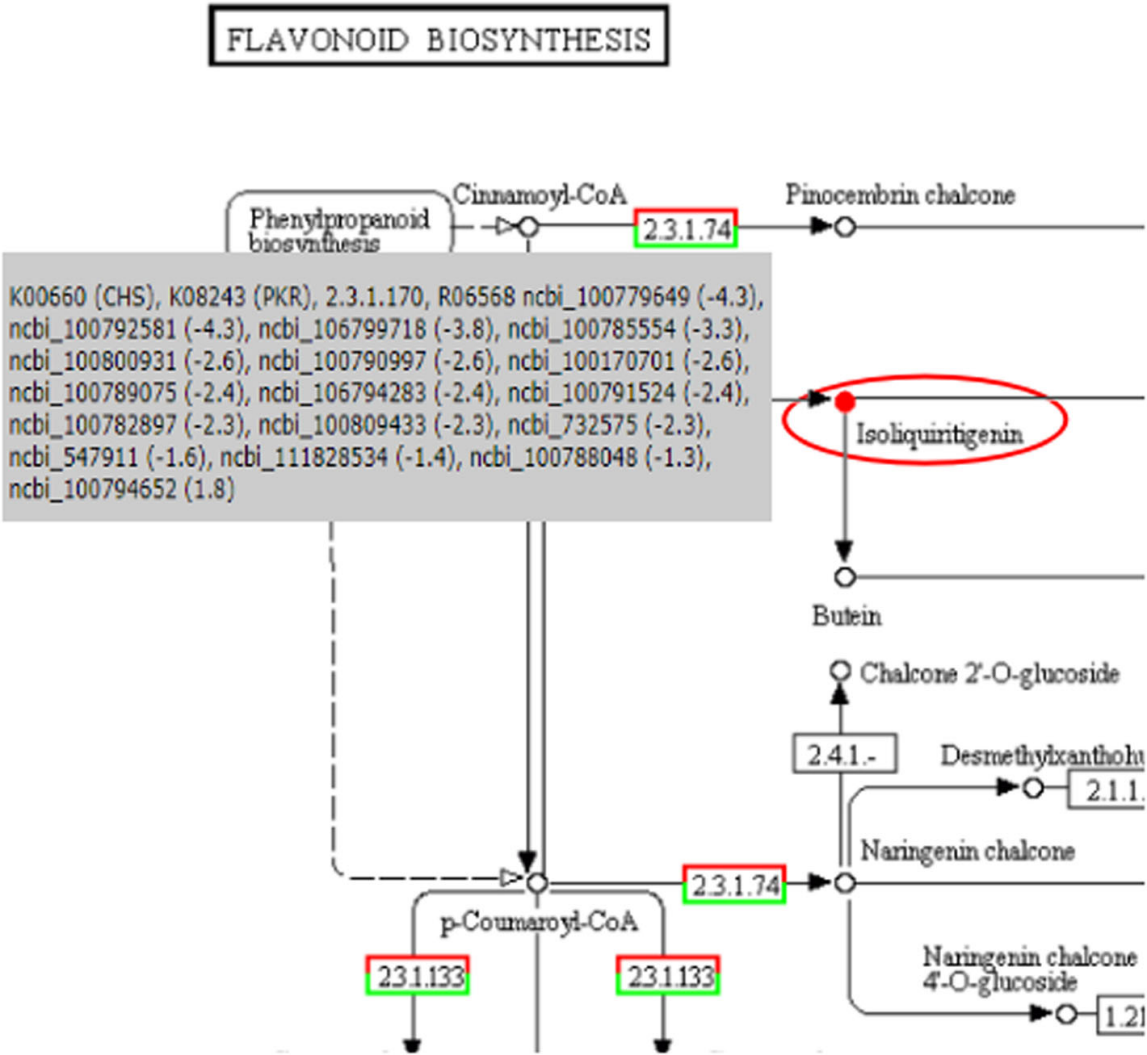
CHS and isoflavone in flavonoid biosynthesis. 2.3.1.74 represent CHS. The red circles mean metabolites are upregulated. The red box means genes upregulated, the green box means genes downregulated,and numbers of CHS genes in NCBI are showed in grey box

Secondary metabolites such as flavonoids could effectively help plants resist diseases, including protecting plants from pathogens, plants auxin transport and mutual recognition and cooperation between plants and microorganisms [5, 12, 29]. In mycorrhizal soybeans, *F. mosseae* strongly promoted the accumulation of flavonoids, such as flavonols, flavone, and anthocyanin. In the CK vs F group, the flavonoid biosynthesis-related genes *CHI*, chalcone synthase (*CHS*), trans-cinnamate 4-monooxygenase, coumaroyl quinate (coumaroyl shikimate) 3′-monooxygenase, caffeoyl-CoA O-methyltransferase, flavonoid 3′-monooxygenase, and shikimate O-hydroxycinnamoyltransferase were upregulated. In the F vs. AF group, most genes were downregulated. In the interaction between plants and pathogens, Isoflavonoids not only act as signalling molecules for the symbiosis of nitrogen-fixing bacteria, but also inhibit the pathogen infection (plant antitoxin) [27].. CHS and CHI are key enzymes in isoflavone synthesis that play crucial roles in plant responses to various pathogens. Their expression efficiency in plants directly affects the isoflavone content. Soybeans contain 9 members of the CHS gene family, from *CHS1* to *CHS9*, and *CHS1* has 2 copies. Although members of this family are highly similar in sequence, they play different roles in plant development. *CHS7* and *CHS8* are primarily involved in isoflavone synthesis and metabolic pathways [54]. There are two primary types of *CHI*, of which *CHI2* only exists in legumes. It can catalyse naringin chalcone and isoliquiritigenin to become naringenin and liquiritigenin, respectively. This finding is consistent with the biosynthesis of isoflavone [31]. In this study, the isoflavones were upregulated after the inoculation with *F. mosseae*, which alleviated the root rot (Fig. 7).

## Discussion

Soybean root rot caused by *F. oxysporum* is a typical destructive soil-borne disease. It has been shown that AMF can enhance plant disease resistance and reduce the harm caused by pathogens. For example, Some authors have shown that all AMF inoculations could reduce the incidence of peanut (*Arachis hypogaea* Linn.) stem rot caused by *Sclerotium rolfsii*, including inoculations of *Glomus etunicatum*, *Glomus mosseae*, *Glomus clarum*, *Glomus caledonium*, *Glomus fasciculatum* and *Gigaspora margarita*; the disease severity was reduced by 37.8% ~ 64.7% under pot experiment conditions, and the disease severity was reduced by 30.6% ~ 47.2% under field testing [37]. Liu designed greenhouse experiments to study the effects of two types of AMF (*G. intraradices* and *G. mosseae*) on the disease resistance of tobacco. The results showed that the incidence and disease index of tobacco cyanosis after inoculating with *G. intraradices* and *G. mosseae* decreased in comparison with the control group without AMF inoculation [23]. Jie found that the DNA level of *F. oxysporum* in the roots and rhizospheric soil samples of soybean plants inoculated with *F. mosseae* decreased significantly, suggesting that *F. mosseae* had a strong inhibitory effect on *F. oxysporum* [16]. At present, there are few studies on the mechanism by which *F. mosseae* alleviates root rot. In this study, soybean HN48 (protein type) was used as the experimental material. To study how the mechanism of *F. mosseae* alleviates root rot, the time gradient sampling method was used to calculate and observe the incidence of soybean root rot; it was found that *F. mosseae* effectively reduced the root rot incidence. This result is consistent with Gao [9].

After being affected by various pathogenic organisms and adversity factors, plants can produce certain defence mechanisms to maintain their normal growth and development [48]. Plant disease resistance is a complex process, and the induction of some defence enzymes (such as POD, PAL, and SOD) is the most important physiological and biochemical resistance mechanism. These enzymes make plants resistant to pathogens by participating in the metabolism of disease-resistant secondary biomass (such as lignin, phenolics, and phytoalexin), or through the metabolism of active oxygen AOS in plants, or by directly inhibiting and killing pathogens [6]. The increase in POD activity can promote the oxidation of phenol to quinone, which is harmful to bacteria. PAL is one of the major enzymes of phenol metabolism, and it affects the synthesis of phenolic compounds. SOD can effectively scavenge oxygen free radicals and protect cells. In this experiment, the *PAL* gene in the phylopanoid biosynthesis pathway was upregulated after being treated with *F. oxysporum*, which was a similar result to that of Li and Ozlem [21, 38]. However, the *PAL* gene was downregulated in the roots of plants treated with *F. mosseae*. We speculated that when the soybean roots were infected with the pathogen, *F. oxysporum* induced the upregulation of *PAL* genes, but after the effect of *F. mosseae*, the continuous cropping disease was relieved and the activity of the *PAL* genes decreased.

Isocitrate dehydrogenase (IDH) can catalyse the oxidative decarboxylation of isocitrate to generate α-ketoglutarate and carbon dioxide and reduce the oxidized NAD(P)^+^ to NAD(P) H, and it is one of the key enzymes in the tricarboxylic acid cycle. Its activity has a great influence on the entire life metabolism of the organism. It has been reported that IDH plays an active role in responding to low temperature, drought and salt stress [20, 25]. In this study, the expression of *IDH* genes in the TCA cycle and glutathione metabolism was upregulated after *F. oxysporum* infection, which was similar to the results of Leterrier et al. in their study on pea (*Pisum sativum* L.) leaves under low temperature stress and mechanical damage, in which the expression levels of *NADP-IDH* were increased by 70 and 40%, respectively [20]. Therefore, we speculated that *IDH* genes play an active role in plant resistance to stress, and protecting cells from adverse factor stress may be an important biological function of *IDH*.

The results in this study indicated that energy metabolism, including glycolysis, the pentose phosphate pathway, TCA and oxidative phosphorylation, were affected by pathogenic fungi. Under stress from the external environment, the primary metabolic function of the pentose phosphate pathway is reduced, and its primary function is to regulate the flow of the carbon source to a secondary metabolism pathway, such as synthetic phytoalexin, lignin and other secondary metabolism pathways [18]. The 6-phosphogluconate dehydrogenase (6-PGDH) pyrazole gets rid of alcohol, and the 2-deoxidation-D-ribose 1-phosphoric acid and 2-deoxidation-1-alpha-D-ribose phosphate in the pentose phosphate pathway were upregulated, and thus we speculated that the primary role of 6-PGDH in plant disease resistance was to contribute five-carbon sugar compounds to the synthesis of phenolic compounds and other resistant substances. In addition, it also provided more NADPH-reducing power to improve plant resistance to pathogenic microbe infections [4, 35]. Notably, this result indicated that *F. mosseae* accelerated the energy metabolism by increasing the production of ATP. Furthermore, the accumulation of proline, 2-amino-3-methyl butyric acid, arginine, glycine, tyrosine, glucose-6-phosphate, and the contents of various organic acids were observed. Compared with the CK and AF groups, there was a general decreasing trend in the levels of most amino acids in the F group, which indicated that the metabolic activity of the soybean roots was inhibited, similar to Van et al.’s study on the metabolic response of *Arabidopsis* (*Arabidopsis thaliana*) roots [46]. AMF can effectively induce the accumulation of amino acids in soybean roots.

Plants respond to pathogens with a series of specific receptors and signals [28]. A cascade of mitogen-activated protein kinases (MAPK) plays a key role in transmitting signals from the outside to the inside of the cell [3]. Studies have shown that in *Arabidopsis*, transcriptional activation of Flg 22-induced receptor like kinase 1(FRK 1), WRK 22 and other downstream targets, thus causing their own defense [33, 40]. In this study, calcium-dependent protein kinase 1, mitogen-activated protein kinase 1 and serine/threonine-protein kinase PBS1 were adjusted significantly in the CK vs. F groups, indicating that *F. oxysporum* can induce the expression of defence-related genes and limits the migration of the pathogen, and thus plants can become resistant to disease.

## Conclusions

In this study, transcriptome and metabolomics analyses were used to study the variations in gene expression patterns and metabolites between continuously cropped soybean roots. Our results revealed that *F. mosseae* significantly reduced the incidence of root rot in continuously cropped soybeans, improving the disease resistance of these plants. In addition, *F. mosseae* could also promote the accumulation of resistance genes such as *PAL, CYP73A, CCR, CHI,* and *IDH* and metabolites such as daidzein, isoliquiritigenin, pyridoxine, isoflavonoid and other metabolites. Our results not only provide theoretical basis for revealing the molecular mechanisms of AMF alleviating soybean root rot, but they also provide a theoretical basis for the development and application of biological agents.

## Methods

### Plant material and inoculation methods

The test soybean seeds (HN48) were purchased from Heilongjiang Academy of Agricultural Sciences (Harbin City, Heilongjiang Province, China), a widely cultivated species in Heilongjiang. The experiment was conducted at the Sugar Industry Research Institute Experimental Station at the Harbin Institute of Technology in Heilongjiang province, China. Soil from soybean continuously cropped for 3 year was used in experiments.

The tested *F. mosseae* strain was isolated by our research group, and it was stored at the Wuhan Institute of Microbiology. China. The strain preservation number was no. CGMCC 3013. Before planting, alfalfa (*Medicago sativa* L.) was used to propagate the *F. mosseae* strain. The tested pathogen was *F. oxysporum*, a dominant fungus in soybean soil in Heilongjiang province, which was provided by the Key Laboratory of Microbiology, Heilongjiang University.

### Sample processing and collection

Experiments were conducted using potted plants. The soil was sterilized for 1 h in a high-pressure sterilizing pot at 121 °C and cooled to room temperature. The surfaces of the soybean seeds were wiped with alcohol, surface-sterilized for 10 min in 5% sodium hypochlorite, and then washed with sterile deionized water four times for 10 min per time. The sterilized seeds were placed in 5 kg of sterilized soil in 50 cm × 60 cm pots. Five seeds were planted in each pot, and three seedlings were kept after they grew out. Three treatments were set up:(1) Group (CK): sowing soybean seeds in sterilized soil; (2) Group (F): sowing soybean seeds in sterilized soil, 44 days after sowing (the soybeans were in the flowering stage), the soybean seeds were inoculated with *F. oxysporum* spore suspension by root injection. (3) Group (AF): 45 g *F. mosseae* inoculants were mixed with the 5 kg of sterilized soil used to grow soybeans, and after 44 days, the soybean seeds were inoculated with *F. oxysporum* spore suspension by root injection. For each treatment, we planted 20 pots. The soybean roots were harvested at high incidence period (60 days after sowing) from the 0–20 cm soil depth. Specifically, three soybean root samples were randomly selected from each treatment and stored in 10 mL centrifuge tubes and kept into a − 80 °C freezer until molecular analysis.

### Determining the incidence index of soybean root rot and the infection rate of AMF

At 44 days after sowing, different root samples were randomly selected every 7 days. The root rot incidence index was counted according to the classification criteria of soybean root rot. The criteria are as follows: Grade 1: sporadic lesions; Grade 2: patches of sporadic lesions; Grade 3: the lesion area accounts for 25% of the total root area; Grade 4: the lesion area accounts for 30% of the total root area, the lesion area is continuous around the stem but the root is not necrotic; Grade 5: the lesion area accounts for more than 50% of the total root area. The calculation formula of root rot incidence index is as follows:

$$ Incidence\ index=\frac{\sum \left( Number\ of\ diease\ plants\  at\  each\ level\times Disease\ level\right)}{Plant\ number\ investigated\times The\ number\ of\ the\ highest\ level}\times 100 $$

Acid fuchsin staining was used to determine the AMF colonization rate [30]. At 44 days after sowing, different root samples were randomly selected every 7 days. The fibrous roots of soybean were washed and cut into a length of 1 cm and placed in a 5 mL centrifuge tube. 50–100 fibrous roots of soybean were randomly selected for staining, slicing and microscopic examination to observe the colonization of AMF in each treatment. The calculation formula of colonization rate is as follows:

$$ Colonization\ rate=\frac{Number\ of\ infected\ root\ egments}{Total\ number\ of\ observed\ root\ egments}\times 100\% $$

All values are expressed as the mean ± SD. The data were analysed by analysis of variance (ANOVA) followed by Tukey’s HSD test using SPSS 23.0 to determine the significance of differences between different treatments (*P* < 0.05).

### RNA extraction and RNA sequencing analysis

RNA extraction and sequencing analysis were performed as described by Yu CJ et al. [56]. The total RNA was extracted by Trizol-based method [32] during a high incidence of soybean root rot (60 days after sowing). After that, the eukaryotic mRNA was enriched with oligonucleotide (dT), and the rRNA was removed with a Ribo Zero™ Magnetic Kit (Epicentre), to enrich the prokaryotic mRNA. Fragmented buffer was used to segment the enriched mRNA, reverse-transcribed into cDNA by random primers, then synthesized the second strand cDNA and purified it with QiaQuick-PCR extraction kit, end-repair, added poly (A) and connected to Illumina sequencing adapters. The ligation products were size-selected by agarose gel electrophoresis, PCR-amplified, and sequenced using an Illumina HiSeq™ 2500 by Gene Denovo Biotechnology Co (Guangzhou, China).

To ensure high data quality, the raw sequence data were filtered to obtain clean data for the subsequent information analysis. In brief, the joint sequence was removed from the sequencing sequence, and reads with all A-bases were removed; reads with N ratios greater than 10% were removed and low-quality reads were removed (the number of bases with a mass value Q ≤ 20 accounted for more than 50% of the entire read). Then, the rRNA of each sample were removed from the reads and located to the reference genome via TopHat2 (version 2.0.3.12) [17], respectively. The alignment parameters were as follows: 1) Maximum read mismatch is 2; 2) The distance between mate-pair reads is 50 bp; 3) The error of distance between mate-pair reads is ±80 bp [15]. A differential gene expression analysis of the three groups was performed using the edge R package (https://www.r-bloggers.com/its-easy-to-cite-and-reference-r/). FDR and log2FC were used to screen the differentially expressed genes. The screening conditions were FDR < 0.05 and |log2FC| > 1. The DEGs were annotated using the Mercator web tool [26] and then loaded onto MapMan software for a functional enrichment analysis [45]. After that, Gene Ontology (GO) and Kyoto Encyclopedia of Genes and Genomics (KEGG) pathway analyses were performed [34, 55].

### Metabolomics analysis

#### Sample preparation and metabolite extraction

All 9 obtained samples (three treatments, three biological replicates) were used for the metabolomics analysis. The freeze-dried samples were crushed using a mixer mill (MM 400, Retsch) with zirconia beads for 1.5 min at 30 Hz. Then, 100 mg of powder was weighed and extracted overnight at 4 °C with 1.0 mL of 70% aqueous methanol containing 0.1 mg/L lidocaine as the internal standard. Following centrifugation at 10000 g for 10 min, the supernatants were absorbed and filtered (SCAA-104, 0.22-μm pore size; ANPEL, Shanghai, China, www.anpel.com.cn/) before LC-MS/MS analysis. The same volume of all samples to be tested were mixed as Quality Control (QC) samples to detect the reproducibility of the results.

#### Liquid chromatography electrospray ionisation tandem mass spectrometry (LC-ESI- MS/MS)

Analysis of the extracted compounds using a LC-ESI-MS/MS system (SCAA-104, 0.22 μm pore size, ANPEL, Shanghai, China, www.anpel.com.cn/; UPLC, Shim-pack UFLC SHIMADZU CBM20A, http://www.shimadzu.com.cn/; MS/MS (Applied Biosystems 4500 QTRAP, http://www.appliedbiosystems.com.cn/). 2 μL of samples were injected onto a Waters ACQUITY UPLC HSS T3 C_18_ column (2.1 mm*100 mm, 1.8 μm) operating at 40 °C and a flow rate of 0.4 mL/min [58]. Compounds were separated using the following gradient: 95:5 Phase A/Phase B at 0 min; 5:95 Phase A/Phase B at 11.0 min; 5:95 Phase A/Phase B at 12.0 min; 95:5 Phase A/Phase B at 12.1 min; 95:5 Phase A/Phase B at 15.0 min [49, 50]. The effluent from the column was connected to an ESI triple quadrupole-linear ion trap (QTRAP)-MS.

LIT and triple quadrupole (QQQ) scans were acquired on a triple quadrupole-linear ion trap mass spectrometer (Q TRAP), AB Sciex QTRAP6500 System, equipped with an ESI-Turbo Ion-Spray interface, operating in a positive ion mode and controlled by Analyst 1.6.1 software (AB Sciex). The operation parameters were set as described by Shahzad M et al. [43]. The monitoring mode was set to multiple-reaction monitoring (MRM).

#### Qualitative and quantitative analysis of metabolites

The qualitative analysis of primary and secondary MS data was performed by searching public databases such as MassBank (http://www.massbank.jp/), KNApSAcK (http://kanaya.naist.jp/KNApSAcK/), HMDB (http://www.hmdb.ca/) [52], MoToDB (http://www.ab.wur.nl/moto/) and METLIN (http://metlin.scripps.edu/index.php) [59] (Zhu ZJ et al., 2013). The repetitive signals of K^+^, Na^+^, NH4^+^, and other large molecular weight species were eliminated during the identification process. The exact m/z of each Q1 (parent ion molecular weight) was obtained to facilitate the identification/annotation of metabolites [53]. The variable importance of the projection (VIP) score of the application orthogonal partial least squares discriminant analysis (OPLS-DA) model was used to rank the best differentiated metabolites between different treatments. a *P* value of t-test of < 0.05 and VIP ≥ 1 were used to screen differential metabolites between samples [49, 50].

## Supplementary information

**Additional file 1: Table S1.** Statistical table showing read filtering information.

**Additional file 2: Table S2.** CK vs. F group metabolic pathway classification.

**Additional file 3: Table S3.** F vs. AF group metabolic pathway classification.

**Additional file 4: Supplementary Figure S1.** A multi-peak diagram of the multi-response monitoring (MRM) mode for metabolite detection (positive ions).

**Additional file 5: Supplementary Figure S2.** OPLS-DA score plots.

## Data Availability

The datasets generated during the current study are available at the NCBI Sequence Read Archive (SRA) under accession number SRP240183.

## Abbreviations

UPLC: Ultra-performance liquid chromatography
MS/MS: Tandem mass spectrometry
DEGs: Differentially expressed genes
AMF: Arbuscular mycorrhizal fungi
GO: Gene Ontology
KEGG: Kyoto encyclopedia of genes and genomics
BP: Biological process
MF: Molecular function
CC: Cellular component
CYP73A: Trans-cinnamate monooxygenase
CCR: Cinnamyl-CoA reductase
PAL: Phenylalanine ammonia lyase
CHI: Chalcone isomerase
CHS: Chalcone synthase
POD: Peroxidase
SOD: Superoxidedismutase
AOS: Activated oxygen species
IDH: Isocitrate dehydrogenase
6-PGDH: 6-phosphogluconate dehydrogenase
MAPK: Mitogen-activated protein kinases
FRK1: Flg22-induced receptor- like kinase 1
