# Supplementary material for: Transcriptome and metabolite profiling reveals the effects of Funneliformis mosseae on the roots of continuously cropped soybeans
Source: BMC Plant Biol. 2020 Oct 21;20:479. doi: 10.1186/s12870-020-02647-2 (PMC7579952; doi:10.1186/s12870-020-02647-2)
Supplement: Supplementary file 1 — Additional file 1: Table S1. Statistical table showing read filtering information. [file 12870_2020_2647_MOESM1_ESM.docx]

Table S1. Statistical table showing read filtering information

| **Sample** | **Number of clean reads** | **Number of high-quality clean reads (%)** |
| --- | --- | --- |
| CK1 | 65191530 | 64202636 (98.48%) |
| CK2 | 78660386 | 77639698 (98.7%) |
| CK3 | 71994002 | 70908028 (98.49%) |
| F1 | 85382434 | 84257600 (98.68%) |
| F2 | 76826168 | 75725428(98.57%) |
| F3 | 81819714 | 80758268 (98.7%) |
| AF1 | 86858988 | 85856264 (98.85%) |
| AF2 | 73588790 | 72202802 (98.12%) |
| AF3 | 86222556 | 85103200 (98.7%) |
