# Supplementary material for: Transcriptome and metabolite profiling reveals the effects of Funneliformis mosseae on the roots of continuously cropped soybeans
Source: BMC Plant Biol. 2020 Oct 21;20:479. doi: 10.1186/s12870-020-02647-2 (PMC7579952; doi:10.1186/s12870-020-02647-2)
Supplement: Supplementary file 2 — Additional file 2: Table S2. CK vs. F group metabolic pathway classification. [file 12870_2020_2647_MOESM2_ESM.docx]

Table S2. CK vs. F group metabolic pathway classification

| Pathway | DEGs genes with pathway annotation | Pvalue | Qvalue | Pathway ID |
| --- | --- | --- | --- | --- |
| [Ribosome](file:///D:/%E5%9F%BA%E8%BF%AA%E5%A5%A5%E6%95%B0%E6%8D%AE/%E6%B5%81%E7%A8%8B%E7%BB%93%E6%9E%9C/%E6%B5%81%E7%A8%8B%E7%BB%93%E6%9E%9C/GDPMR4419-Glycine_max-RNAseq-result/7.GroupsDifferentialExpression/Enrichment/KO/CK-vs-F.htm#gene1) | 304 (13.04%) | 0.000000 | 0.000000 | ko03010 |
| Phenylpropanoid biosynthesis | 155 (6.65%) | 0.000000 | 0.000000 | ko00940 |
| [Biosynthesis of secondary metabolites](file:///D:/%E5%9F%BA%E8%BF%AA%E5%A5%A5%E6%95%B0%E6%8D%AE/%E6%B5%81%E7%A8%8B%E7%BB%93%E6%9E%9C/%E6%B5%81%E7%A8%8B%E7%BB%93%E6%9E%9C/GDPMR4419-Glycine_max-RNAseq-result/7.GroupsDifferentialExpression/Enrichment/KO/CK-vs-F.htm#gene3) | 669 (28.69%) | 0.000000 | 0.000000 | ko01110 |
| [Ribosome biogenesis in eukaryotes](file:///D:/%E5%9F%BA%E8%BF%AA%E5%A5%A5%E6%95%B0%E6%8D%AE/%E6%B5%81%E7%A8%8B%E7%BB%93%E6%9E%9C/%E6%B5%81%E7%A8%8B%E7%BB%93%E6%9E%9C/GDPMR4419-Glycine_max-RNAseq-result/7.GroupsDifferentialExpression/Enrichment/KO/CK-vs-F.htm#gene4) | 88 (3.77%) | 0.000000 | 0.000000 | ko03008 |
| [Flavonoid biosynthesis](file:///D:/%E5%9F%BA%E8%BF%AA%E5%A5%A5%E6%95%B0%E6%8D%AE/%E6%B5%81%E7%A8%8B%E7%BB%93%E6%9E%9C/%E6%B5%81%E7%A8%8B%E7%BB%93%E6%9E%9C/GDPMR4419-Glycine_max-RNAseq-result/7.GroupsDifferentialExpression/Enrichment/KO/CK-vs-F.htm#gene5) | 40 (1.72%) | 0.000025 | 0.000661 | ko00941 |
| [Glycine, serine and threonine metabolism](file:///D:/%E5%9F%BA%E8%BF%AA%E5%A5%A5%E6%95%B0%E6%8D%AE/%E6%B5%81%E7%A8%8B%E7%BB%93%E6%9E%9C/%E6%B5%81%E7%A8%8B%E7%BB%93%E6%9E%9C/GDPMR4419-Glycine_max-RNAseq-result/7.GroupsDifferentialExpression/Enrichment/KO/CK-vs-F.htm#gene6) | 51 (2.19%) | 0.000440 | 0.008734 | ko00260 |
| [Metabolic pathways](file:///D:/%E5%9F%BA%E8%BF%AA%E5%A5%A5%E6%95%B0%E6%8D%AE/%E6%B5%81%E7%A8%8B%E7%BB%93%E6%9E%9C/%E6%B5%81%E7%A8%8B%E7%BB%93%E6%9E%9C/GDPMR4419-Glycine_max-RNAseq-result/7.GroupsDifferentialExpression/Enrichment/KO/CK-vs-F.htm#gene9) | 990 (42.45%) | 0.001923 | 0.025440 | ko01100 |
| [Phenylalanine metabolism](file:///D:/%E5%9F%BA%E8%BF%AA%E5%A5%A5%E6%95%B0%E6%8D%AE/%E6%B5%81%E7%A8%8B%E7%BB%93%E6%9E%9C/%E6%B5%81%E7%A8%8B%E7%BB%93%E6%9E%9C/GDPMR4419-Glycine_max-RNAseq-result/7.GroupsDifferentialExpression/Enrichment/KO/CK-vs-F.htm#gene10) | 32 (1.37%) | 0.001942 | 0.025440 | ko00360 |
| Isoflavonoid biosynthesis | 16 (0.69%) | 0.005253 | 0.049156 | ko00943 |
| Arachidonic acid metabolism | 16 (0.69%) | 0.007239 | 0.063223 | ko00590 |
| Biosynthesis of amino acids | 124 (5.32%) | 0.110137 | 0.437210 | ko01230 |
| [Biosynthesis of antibiotics](file:///D:/%E5%9F%BA%E8%BF%AA%E5%A5%A5%E6%95%B0%E6%8D%AE/%E6%B5%81%E7%A8%8B%E7%BB%93%E6%9E%9C/%E6%B5%81%E7%A8%8B%E7%BB%93%E6%9E%9C/GDPMR4419-Glycine_max-RNAseq-result/7.GroupsDifferentialExpression/Enrichment/KO/CK-vs-F.htm#gene35) | 231 (9.91%) | 0.138263 | 0.517499 | ko01130 |
| Circadian rhythm - plant | 33 (1.42%) | 0.158729 | 0.561987 | ko04712 |
| Carbon metabolism | 137 (5.87%) | 0.171690 | 0.576703 | ko01200 |
| [Plant-pathogen interaction](file:///D:/%E5%9F%BA%E8%BF%AA%E5%A5%A5%E6%95%B0%E6%8D%AE/%E6%B5%81%E7%A8%8B%E7%BB%93%E6%9E%9C/%E6%B5%81%E7%A8%8B%E7%BB%93%E6%9E%9C/GDPMR4419-Glycine_max-RNAseq-result/7.GroupsDifferentialExpression/Enrichment/KO/CK-vs-F.htm#gene42) | 104 (4.46%) | 0.206435 | 0.638910 | ko04626 |
| [Glutathione metabolism](file:///D:/%E5%9F%BA%E8%BF%AA%E5%A5%A5%E6%95%B0%E6%8D%AE/%E6%B5%81%E7%A8%8B%E7%BB%93%E6%9E%9C/%E6%B5%81%E7%A8%8B%E7%BB%93%E6%9E%9C/GDPMR4419-Glycine_max-RNAseq-result/7.GroupsDifferentialExpression/Enrichment/KO/CK-vs-F.htm#gene43) | 48 (2.06%) | 0.209719 | 0.638910 | ko00480 |
| [Sulfur metabolism](file:///D:/%E5%9F%BA%E8%BF%AA%E5%A5%A5%E6%95%B0%E6%8D%AE/%E6%B5%81%E7%A8%8B%E7%BB%93%E6%9E%9C/%E6%B5%81%E7%A8%8B%E7%BB%93%E6%9E%9C/GDPMR4419-Glycine_max-RNAseq-result/7.GroupsDifferentialExpression/Enrichment/KO/CK-vs-F.htm#gene45) | 20 (0.86%) | 0.233922 | 0.662490 | ko00920 |
| [Citrate cycle (TCA cycle)](file:///D:/%E5%9F%BA%E8%BF%AA%E5%A5%A5%E6%95%B0%E6%8D%AE/%E6%B5%81%E7%A8%8B%E7%BB%93%E6%9E%9C/%E6%B5%81%E7%A8%8B%E7%BB%93%E6%9E%9C/GDPMR4419-Glycine_max-RNAseq-result/7.GroupsDifferentialExpression/Enrichment/KO/CK-vs-F.htm#gene51) | 30 (1.29%) | 0.264472 | 0.677593 | ko00020 |
| [Starch and sucrose metabolism](file:///D:/%E5%9F%BA%E8%BF%AA%E5%A5%A5%E6%95%B0%E6%8D%AE/%E6%B5%81%E7%A8%8B%E7%BB%93%E6%9E%9C/%E6%B5%81%E7%A8%8B%E7%BB%93%E6%9E%9C/GDPMR4419-Glycine_max-RNAseq-result/7.GroupsDifferentialExpression/Enrichment/KO/CK-vs-F.htm#gene63) | 116 (4.97%) | 0.486248 | 0.983630 | ko00500 |
| [Plant hormone signal transduction](file:///D:/%E5%9F%BA%E8%BF%AA%E5%A5%A5%E6%95%B0%E6%8D%AE/%E6%B5%81%E7%A8%8B%E7%BB%93%E6%9E%9C/%E6%B5%81%E7%A8%8B%E7%BB%93%E6%9E%9C/GDPMR4419-Glycine_max-RNAseq-result/7.GroupsDifferentialExpression/Enrichment/KO/CK-vs-F.htm#gene99) | 156 (6.69%) | 0.913704 | 1.000000 | ko04075 |
| Photosynthesis | 19 (0.81%) | 0.989746 | 1.000000 | ko00195 |
| [DNA replication](file:///D:/%E5%9F%BA%E8%BF%AA%E5%A5%A5%E6%95%B0%E6%8D%AE/%E6%B5%81%E7%A8%8B%E7%BB%93%E6%9E%9C/%E6%B5%81%E7%A8%8B%E7%BB%93%E6%9E%9C/GDPMR4419-Glycine_max-RNAseq-result/7.GroupsDifferentialExpression/Enrichment/KO/CK-vs-F.htm#gene125) | 20 (0.86%) | 0.999998 | 1.000000 | ko03030 |
| [Ubiquitin mediated proteolysis](file:///D:/%E5%9F%BA%E8%BF%AA%E5%A5%A5%E6%95%B0%E6%8D%AE/%E6%B5%81%E7%A8%8B%E7%BB%93%E6%9E%9C/%E6%B5%81%E7%A8%8B%E7%BB%93%E6%9E%9C/GDPMR4419-Glycine_max-RNAseq-result/7.GroupsDifferentialExpression/Enrichment/KO/CK-vs-F.htm#gene131) | 19 (0.81%) | 1.000000 | 1.000000 | ko04120 |
