# Supplementary material for: Transcriptome and metabolite profiling reveals the effects of Funneliformis mosseae on the roots of continuously cropped soybeans
Source: BMC Plant Biol. 2020 Oct 21;20:479. doi: 10.1186/s12870-020-02647-2 (PMC7579952; doi:10.1186/s12870-020-02647-2)
Supplement: Supplementary file 3 — Additional file 3: Table S3. F vs. AF group metabolic pathway classification. [file 12870_2020_2647_MOESM3_ESM.docx]

Table S3. F vs. AF group metabolic pathway classification

| Pathway | DEGs genes with pathway annotation | Pvalue | Qvalue | Pathway ID |
| --- | --- | --- | --- | --- |
| Ribosome | 392 (14.35%) | 0.000000 | 0.000000 | ko03010 |
| [Biosynthesis of secondary metabolites](" \o "click to view genes) | 758 (27.76%) | 0.000000 | 0.000000 | ko01110 |
| Phenylpropanoid biosynthesis | 148 (5.42%) | 0.000000 | 0.000008 | ko00940 |
| [Microbial metabolism in diverse environments](" \o "click to view genes) | 257 (9.41%) | 0.000013 | 0.000342 | ko01120 |
| [Glycine, serine and threonine metabolism](" \o "click to view genes) | 62 (2.27%) | 0.000018 | 0.000387 | ko00260 |
| [Biosynthesis of amino acids](" \o "click to view genes) | 171 (6.26%) | 0.000032 | 0.000603 | ko01230 |
| [Biosynthesis of antibiotics](" \o "click to view genes) | 303 (11.09%) | 0.000115 | 0.001898 | ko01130 |
| [Glyoxylate and dicarboxylate metabolism](file:///H:/%E5%9F%BA%E8%BF%AA%E5%A5%A5%E6%95%B0%E6%8D%AE/%E5%9F%BA%E8%BF%AA%E5%A5%A5%E6%95%B0%E6%8D%AE/%E6%B5%81%E7%A8%8B%E7%BB%93%E6%9E%9C/%E6%B5%81%E7%A8%8B%E7%BB%93%E6%9E%9C/GDPMR4419-Glycine_max-RNAseq-result/7.GroupsDifferentialExpression/Enrichment/KO/F-vs-AF.htm#gene10) | 60 (2.2%) | 0.000201 | 0.002467 | ko00630 |
| [Flavonoid biosynthesis](file:///H:/%E5%9F%BA%E8%BF%AA%E5%A5%A5%E6%95%B0%E6%8D%AE/%E5%9F%BA%E8%BF%AA%E5%A5%A5%E6%95%B0%E6%8D%AE/%E6%B5%81%E7%A8%8B%E7%BB%93%E6%9E%9C/%E6%B5%81%E7%A8%8B%E7%BB%93%E6%9E%9C/GDPMR4419-Glycine_max-RNAseq-result/7.GroupsDifferentialExpression/Enrichment/KO/F-vs-AF.htm#gene11) | 42 (1.54%) | 0.000206 | 0.002467 | ko00941 |
| [Carbon metabolism](file:///H:/%E5%9F%BA%E8%BF%AA%E5%A5%A5%E6%95%B0%E6%8D%AE/%E5%9F%BA%E8%BF%AA%E5%A5%A5%E6%95%B0%E6%8D%AE/%E6%B5%81%E7%A8%8B%E7%BB%93%E6%9E%9C/%E6%B5%81%E7%A8%8B%E7%BB%93%E6%9E%9C/GDPMR4419-Glycine_max-RNAseq-result/7.GroupsDifferentialExpression/Enrichment/KO/F-vs-AF.htm#gene12) | 185 (6.77%) | 0.000298 | 0.003277 | ko01200 |
| [Alanine, aspartate and glutamate metabolism](file:///H:/%E5%9F%BA%E8%BF%AA%E5%A5%A5%E6%95%B0%E6%8D%AE/%E5%9F%BA%E8%BF%AA%E5%A5%A5%E6%95%B0%E6%8D%AE/%E6%B5%81%E7%A8%8B%E7%BB%93%E6%9E%9C/%E6%B5%81%E7%A8%8B%E7%BB%93%E6%9E%9C/GDPMR4419-Glycine_max-RNAseq-result/7.GroupsDifferentialExpression/Enrichment/KO/F-vs-AF.htm#gene13) | 44 (1.61%) | 0.000865 | 0.008782 | ko00250 |
| [Cysteine and methionine metabolism](file:///H:/%E5%9F%BA%E8%BF%AA%E5%A5%A5%E6%95%B0%E6%8D%AE/%E5%9F%BA%E8%BF%AA%E5%A5%A5%E6%95%B0%E6%8D%AE/%E6%B5%81%E7%A8%8B%E7%BB%93%E6%9E%9C/%E6%B5%81%E7%A8%8B%E7%BB%93%E6%9E%9C/GDPMR4419-Glycine_max-RNAseq-result/7.GroupsDifferentialExpression/Enrichment/KO/F-vs-AF.htm#gene14) | 79 (2.89%) | 0.000971 | 0.009156 | ko00270 |
| [Isoflavonoid biosynthesis](file:///H:/%E5%9F%BA%E8%BF%AA%E5%A5%A5%E6%95%B0%E6%8D%AE/%E5%9F%BA%E8%BF%AA%E5%A5%A5%E6%95%B0%E6%8D%AE/%E6%B5%81%E7%A8%8B%E7%BB%93%E6%9E%9C/%E6%B5%81%E7%A8%8B%E7%BB%93%E6%9E%9C/GDPMR4419-Glycine_max-RNAseq-result/7.GroupsDifferentialExpression/Enrichment/KO/F-vs-AF.htm#gene15) | 19 (0.7%) | 0.001415 | 0.012454 | ko00943 |
| [Metabolic pathways](file:///H:/%E5%9F%BA%E8%BF%AA%E5%A5%A5%E6%95%B0%E6%8D%AE/%E5%9F%BA%E8%BF%AA%E5%A5%A5%E6%95%B0%E6%8D%AE/%E6%B5%81%E7%A8%8B%E7%BB%93%E6%9E%9C/%E6%B5%81%E7%A8%8B%E7%BB%93%E6%9E%9C/GDPMR4419-Glycine_max-RNAseq-result/7.GroupsDifferentialExpression/Enrichment/KO/F-vs-AF.htm#gene24) | 1133 (41.49%) | 0.022269 | 0.122482 | ko01100 |
| [Citrate cycle (TCA cycle)](file:///H:/%E5%9F%BA%E8%BF%AA%E5%A5%A5%E6%95%B0%E6%8D%AE/%E5%9F%BA%E8%BF%AA%E5%A5%A5%E6%95%B0%E6%8D%AE/%E6%B5%81%E7%A8%8B%E7%BB%93%E6%9E%9C/%E6%B5%81%E7%A8%8B%E7%BB%93%E6%9E%9C/GDPMR4419-Glycine_max-RNAseq-result/7.GroupsDifferentialExpression/Enrichment/KO/F-vs-AF.htm#gene26) | 41 (1.5%) | 0.027902 | 0.141658 | ko00020 |
| [Tyrosine metabolism](file:///H:/%E5%9F%BA%E8%BF%AA%E5%A5%A5%E6%95%B0%E6%8D%AE/%E5%9F%BA%E8%BF%AA%E5%A5%A5%E6%95%B0%E6%8D%AE/%E6%B5%81%E7%A8%8B%E7%BB%93%E6%9E%9C/%E6%B5%81%E7%A8%8B%E7%BB%93%E6%9E%9C/GDPMR4419-Glycine_max-RNAseq-result/7.GroupsDifferentialExpression/Enrichment/KO/F-vs-AF.htm#gene31) | 36 (1.32%) | 0.054906 | 0.233795 | ko00350 |
| [Glutathione metabolism](file:///H:/%E5%9F%BA%E8%BF%AA%E5%A5%A5%E6%95%B0%E6%8D%AE/%E5%9F%BA%E8%BF%AA%E5%A5%A5%E6%95%B0%E6%8D%AE/%E6%B5%81%E7%A8%8B%E7%BB%93%E6%9E%9C/%E6%B5%81%E7%A8%8B%E7%BB%93%E6%9E%9C/GDPMR4419-Glycine_max-RNAseq-result/7.GroupsDifferentialExpression/Enrichment/KO/F-vs-AF.htm#gene33) | 60 (2.2%) | 0.063369 | 0.253477 | ko00480 |
| [Valine, leucine and isoleucine degradation](file:///H:/%E5%9F%BA%E8%BF%AA%E5%A5%A5%E6%95%B0%E6%8D%AE/%E5%9F%BA%E8%BF%AA%E5%A5%A5%E6%95%B0%E6%8D%AE/%E6%B5%81%E7%A8%8B%E7%BB%93%E6%9E%9C/%E6%B5%81%E7%A8%8B%E7%BB%93%E6%9E%9C/GDPMR4419-Glycine_max-RNAseq-result/7.GroupsDifferentialExpression/Enrichment/KO/F-vs-AF.htm#gene37) | 36 (1.32%) | 0.080727 | 0.287998 | ko00280 |
| [Plant-pathogen interaction](file:///H:/%E5%9F%BA%E8%BF%AA%E5%A5%A5%E6%95%B0%E6%8D%AE/%E5%9F%BA%E8%BF%AA%E5%A5%A5%E6%95%B0%E6%8D%AE/%E6%B5%81%E7%A8%8B%E7%BB%93%E6%9E%9C/%E6%B5%81%E7%A8%8B%E7%BB%93%E6%9E%9C/GDPMR4419-Glycine_max-RNAseq-result/7.GroupsDifferentialExpression/Enrichment/KO/F-vs-AF.htm#gene42) | 124 (4.54%) | 0.122211 | 0.384092 | ko04626 |
| [Carotenoid biosynthesis](file:///H:/%E5%9F%BA%E8%BF%AA%E5%A5%A5%E6%95%B0%E6%8D%AE/%E5%9F%BA%E8%BF%AA%E5%A5%A5%E6%95%B0%E6%8D%AE/%E6%B5%81%E7%A8%8B%E7%BB%93%E6%9E%9C/%E6%B5%81%E7%A8%8B%E7%BB%93%E6%9E%9C/GDPMR4419-Glycine_max-RNAseq-result/7.GroupsDifferentialExpression/Enrichment/KO/F-vs-AF.htm#gene55) | 22 (0.81%) | 0.297510 | 0.709359 | ko00906 |
| [Purine metabolism](file:///H:/%E5%9F%BA%E8%BF%AA%E5%A5%A5%E6%95%B0%E6%8D%AE/%E5%9F%BA%E8%BF%AA%E5%A5%A5%E6%95%B0%E6%8D%AE/%E6%B5%81%E7%A8%8B%E7%BB%93%E6%9E%9C/%E6%B5%81%E7%A8%8B%E7%BB%93%E6%9E%9C/GDPMR4419-Glycine_max-RNAseq-result/7.GroupsDifferentialExpression/Enrichment/KO/F-vs-AF.htm#gene56) | 94 (3.44%) | 0.300940 | 0.709359 | ko00230 |
| [Pentose phosphate pathway](file:///H:/%E5%9F%BA%E8%BF%AA%E5%A5%A5%E6%95%B0%E6%8D%AE/%E5%9F%BA%E8%BF%AA%E5%A5%A5%E6%95%B0%E6%8D%AE/%E6%B5%81%E7%A8%8B%E7%BB%93%E6%9E%9C/%E6%B5%81%E7%A8%8B%E7%BB%93%E6%9E%9C/GDPMR4419-Glycine_max-RNAseq-result/7.GroupsDifferentialExpression/Enrichment/KO/F-vs-AF.htm#gene57) | 33 (1.21%) | 0.348773 | 0.783426 | ko00030 |
| [Fatty acid degradation](file:///H:/%E5%9F%BA%E8%BF%AA%E5%A5%A5%E6%95%B0%E6%8D%AE/%E5%9F%BA%E8%BF%AA%E5%A5%A5%E6%95%B0%E6%8D%AE/%E6%B5%81%E7%A8%8B%E7%BB%93%E6%9E%9C/%E6%B5%81%E7%A8%8B%E7%BB%93%E6%9E%9C/GDPMR4419-Glycine_max-RNAseq-result/7.GroupsDifferentialExpression/Enrichment/KO/F-vs-AF.htm#gene46#gene61) | 31 (1.14%) | 0.394654 | 0.842927 | ko00071 |
| [RNA transport](file:///H:/%E5%9F%BA%E8%BF%AA%E5%A5%A5%E6%95%B0%E6%8D%AE/%E5%9F%BA%E8%BF%AA%E5%A5%A5%E6%95%B0%E6%8D%AE/%E6%B5%81%E7%A8%8B%E7%BB%93%E6%9E%9C/%E6%B5%81%E7%A8%8B%E7%BB%93%E6%9E%9C/GDPMR4419-Glycine_max-RNAseq-result/7.GroupsDifferentialExpression/Enrichment/KO/F-vs-AF.htm#gene46#gene103) | 80 (2.93%) | 0.908552 | 1.000000 | ko03013 |
| [Protein processing in endoplasmic reticulum](file:///H:/%E5%9F%BA%E8%BF%AA%E5%A5%A5%E6%95%B0%E6%8D%AE/%E5%9F%BA%E8%BF%AA%E5%A5%A5%E6%95%B0%E6%8D%AE/%E6%B5%81%E7%A8%8B%E7%BB%93%E6%9E%9C/%E6%B5%81%E7%A8%8B%E7%BB%93%E6%9E%9C/GDPMR4419-Glycine_max-RNAseq-result/7.GroupsDifferentialExpression/Enrichment/KO/F-vs-AF.htm#gene46#gene125) | 82 (3%) | 0.999998 | 1.000000 | ko04141 |
