# Supplementary material for: Transcriptome and metabolite profiling reveals the effects of Funneliformis mosseae on the roots of continuously cropped soybeans
Source: BMC Plant Biol. 2020 Oct 21;20:479. doi: 10.1186/s12870-020-02647-2 (PMC7579952; doi:10.1186/s12870-020-02647-2)
Supplement: Supplementary file 4 — Additional file 4: Supplementary Figure S1. A multi-peak diagram of the multi-response monitoring (MRM) mode for metabolite detection (positive ions). [file 12870_2020_2647_MOESM4_ESM.docx]

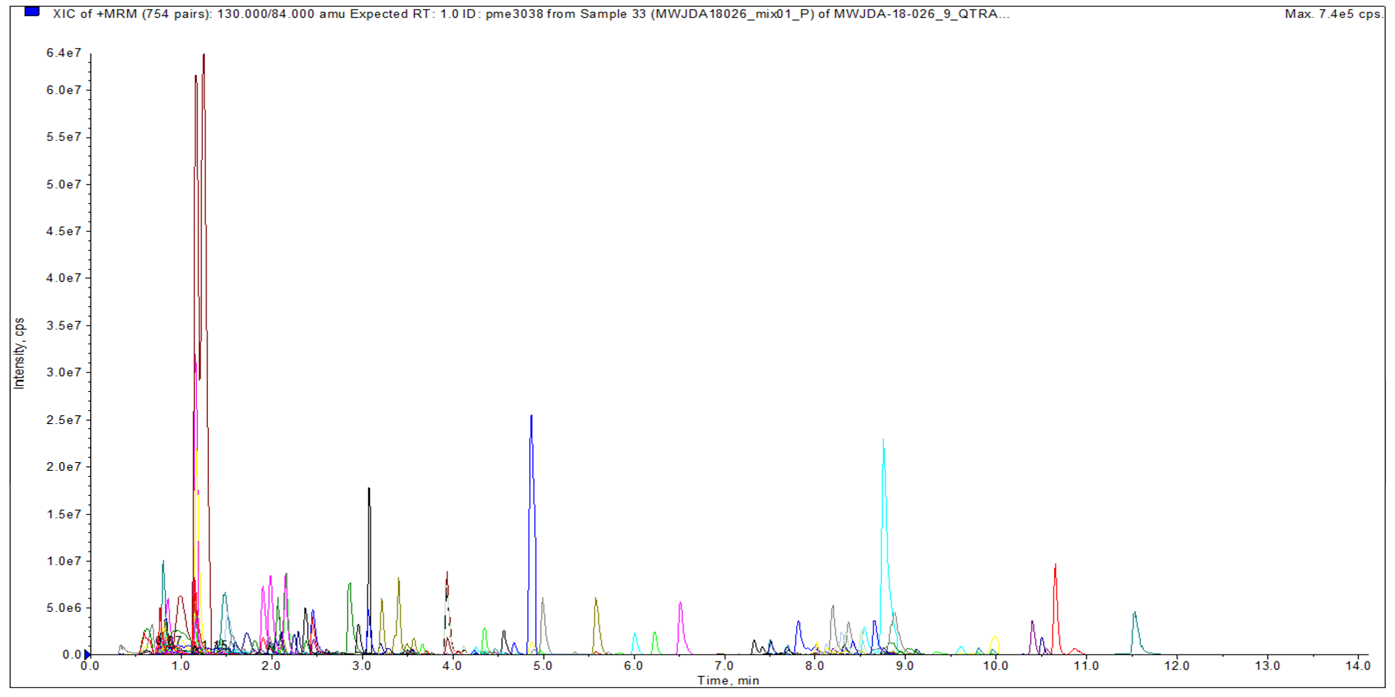


Supplementary Figure S1. A multi-peak diagram of the multi-response monitoring (MRM) mode for metabolite detection (positive ions).
