# Supplementary figures and images for: Transcriptome and metabolite profiling reveals the effects of Funneliformis mosseae on the roots of continuously cropped soybeans
Source: BMC Plant Biol. 2020 Oct 21;20:479. doi: 10.1186/s12870-020-02647-2 (PMC7579952; doi:10.1186/s12870-020-02647-2)

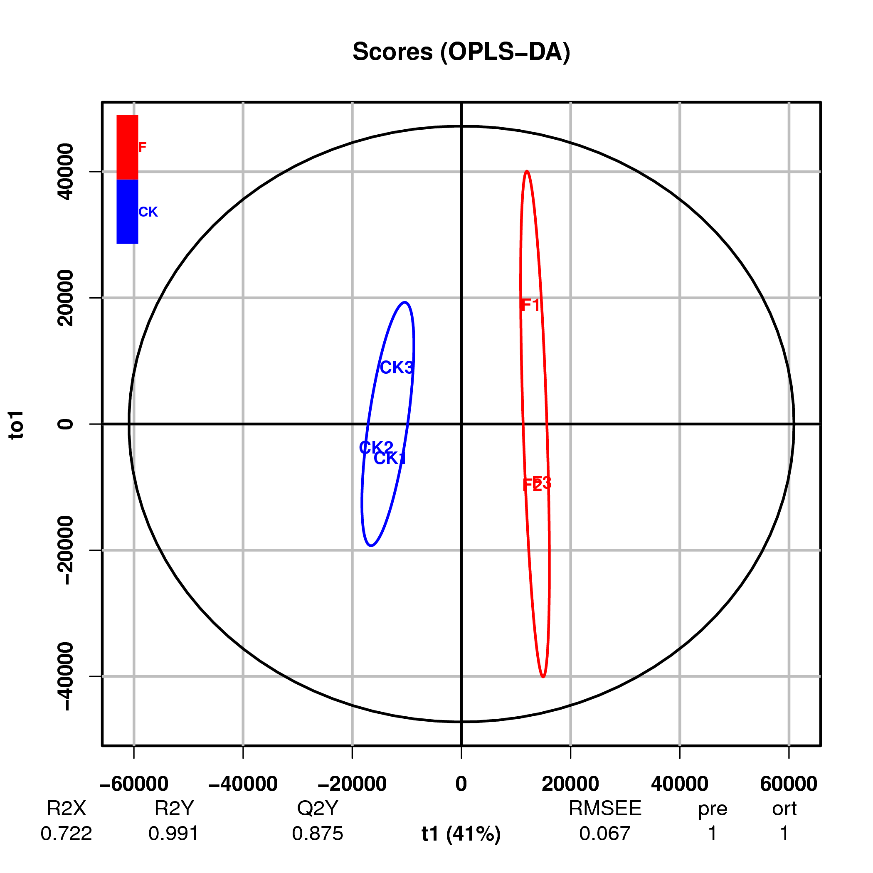


**A**


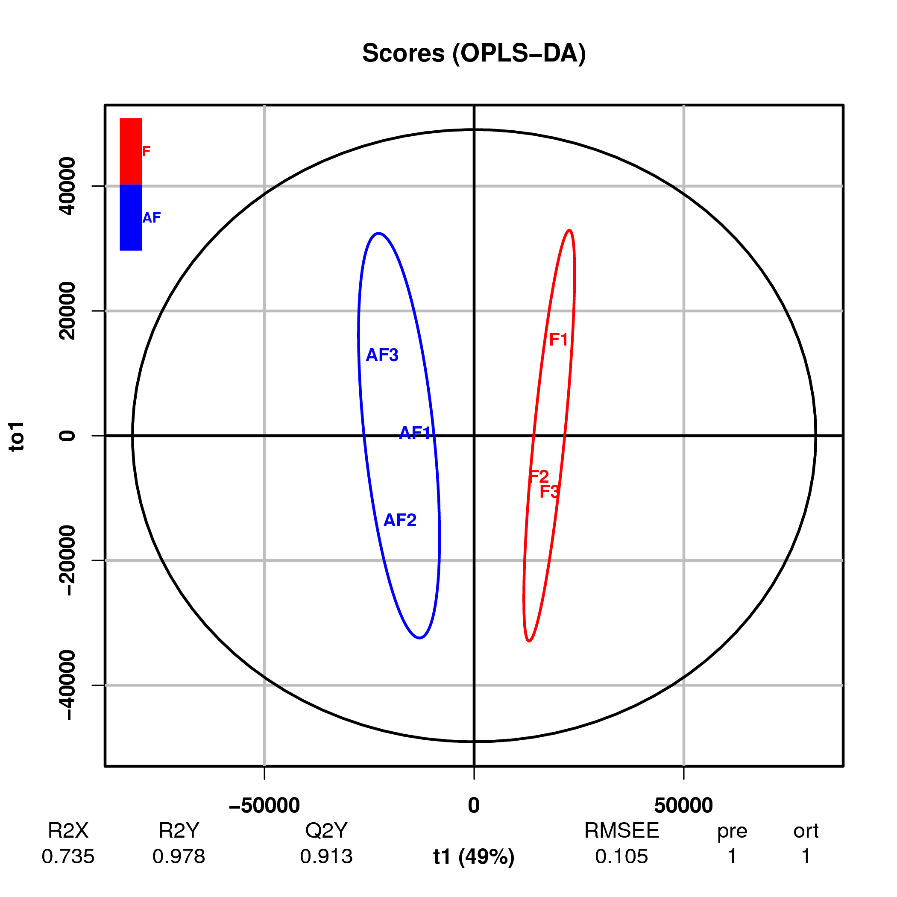


**B**

Supplementary Figure S2 . OPLS-DA score plots

Supplement: Supplementary file 5 — Additional file 5: Supplementary Figure S2. OPLS-DA score plots. [file 12870_2020_2647_MOESM5_ESM.docx]
